# How to prevent non-communicable diseases? - A continuous need for a better understanding of the role of nutritional factors through scientific research

**DOI:** 10.1038/s41430-021-00997-0

**Published:** 2021-10-28

**Authors:** Jaakko Tuomilehto

**Affiliations:** 1 Public Health Promotion Unit, Finnish Institute for Health and Welfare, Helsinki, Finland; 2 Department of Public Health, University of Helsinki, Helsinki, Finland; 3 Saudi Diabetes Research Group, King Abdulaziz University, Jeddah, Saudi Arabia; 4 Department of International Health, National School of Public Health, Instituto de Salud Carlos III, Madrid, Spain

**Keywords:** Epidemiology, Preventive medicine



I was born on a farm in Finland in 1946, during the “baby-boom” after the 2nd World War when life was not easy in a country that was considered having lost the war against our big neighbour that had attacked us. My family has owned the farm from its very start in the late thirteenth century and now I own it. After the war, we had food enough since we had animals and could grow many types of food. Nevertheless, food did not come to the table without major physical effort, I learned that firsthand! Today, we would call many of these foods—rye, barley, oats, potato, tomato, cucumber, lettuce, many sorts of roots, berries, etc.—“functional” healthy foods. However, we also had butter and cheese from the local dairy at a discounted producer price, as all dairy farmers in Finland had at that time. Today, on my farm I grow blackcurrants that are scientifically proven to be very healthy [1]. In comparison with many other berries, the blackcurrant is rich in anthocyanins and proanthocyanins [2], and soluble fibre among many other healthy constituents, thus making it interesting for health-conscious people and industry. Studies have shown that various blackcurrant products lower blood cholesterol, blood pressure and postprandial glucose [1].

My mother was a leader of the local housewife’s organization and was highly appreciated for her knowledge and skills in cooking, and nutritional knowledge obtained from various sources. I had the possibility to watch her in our kitchen and participate as a child in many small jobs with food preparation. This sparked my interest in issues related to nutrition and at University with my roommate, we would cook our own dinner every day. Ever since I have loved preparing meals and am eager to try out new ideas in cooking.

One interesting thing in my own diet history occurred: I didn’t like to drink milk from our own cows, instead actively avoided it. Only much later, when I wondered why my children had stomach problems when they were small, did I realize that this could be due to lactose intolerance, which is more common in Finland than in other parts of Europe. So, we found out that I and most of my children are lactose intolerance. The genetic background of lactose intolerance was identified by researchers at my institute in the 1990s [3]. It became clear that lactose intolerance was the wild (original) genotype among humans, and that the tolerance to lactose many people had, had gradually developed in people during centuries of using cow’s milk.

## The start: cardiovascular disease (CVD) prevention

My original research related to diet and nutrition started from a different aspect of dairy products. It was related to the high serum cholesterol level among Finnish men, first described by the Seven Countries’ Study researchers in the late 1950s and early 1960s [4] by the team of Ancel Keys whose collaborator in Finland was Prof. Martti Karvonen. Finland ranked the highest for the serum cholesterol concentration and coronary heart disease occurrence. The world record of coronary heart disease mortality was found among middle-aged men in the province of North Karelia in eastern Finland. The lumberjacks in eastern Finland were slim and physically very fit. Their work that was physically extremely demanding, so they were consuming 7000–8000 kcal/day. Much of it came from saturated fat. With the support of the regional administration of the province of North Karelia, the Finnish Ministry of Health and several other organisations our team comprising young medical people Prof. Pekka Puska as the Principal Investigator initiated the study called the North Karelia Project that became the “mother” of community-based cardiovascular (CVD) and non-communicable disease (NCD) prevention actions [5, 6].

Investing time and effort entirely on the North Karelia Project as young physicians, we were warned by many Finnish colleagues that we may ruin our future medical careers by taking such a high-risk stake that nobody had tried before. On the other hand, officers from the World Health Organisation and famous international CVD epidemiologists gave us very strong support. When we asked their advice at the beginning as to how we should proceed with such a community-based CVD prevention project, the answer from them was: “Sorry, we do not know, but since no previous example exists, do it and then tell all of us how it worked out,”. We worked with health personnel, but even more importantly with several community organisations, commercial companies, local dairy, housewife associations, shops, etc. to promote healthy, low-fat nutrition. The baseline survey in 1972 confirmed high levels of CVD risk factors; the mean serum cholesterol in men aged 25–59 years was 7.2 mmol/L [7]! The community-based intervention developed successfully. A good example is the following: when we realised that nobody consumed skimmed milk in North Karelia, we asked people why not? They answered that skimmed milk is not available in the shops. Then we asked shop owners why they don’t they sell skimmed milk, the answer was that the local dairy is not producing it. Then we asked the local dairy why they don’t produce skimmed milk, the answer was that shops are not asking for it. A vicious circle that we could easily solve, and the dairy started to produce skimmed milk happily, since then they could sell products from milk fat elsewhere and have additional profits. Many foreign people were surprised that we had such a good collaboration with the local dairy in our efforts to prevent CVD.

The story of the North Karelia Project ended well. Many aspects of lifestyle, especially diet have changed dramatically in eastern Finland, namely by a very significant decrease in serum cholesterol and blood pressure [8, 9]. Today, coronary heart disease (CHD) mortality in North Karelian men aged <65 years is ~90% lower than in the early 1970s [10].

My main responsibility in the North Karelia Project was related to hypertension. At the time of the start of the North Karelia Project favourable results from the first controlled trials using available antihypertensive drugs from the Veterans Administration trial were published around 1970 [11]. This gave us a scientific basis for the development of a community-based hypertension control programme in North Karelia that I developed and implemented with the local primary care workers [12]. One of the most important components of the hypertension control programme was to train nurses from each local community to work with local primary care physicians in the management of people with hypertension and to organize a screening for hypertension. This model soon became a national guideline for hypertension care in Finland. Our data on monitoring indicators of hypertension care has shown marked improvements in awareness, treatment and control of high blood pressure in North Karelia and other parts of Finland [9].

In an observational prospective study of middle-aged men we found that elevated serum ferritin was a strong risk factor for acute myocardial infarction in multivariate models. This association was stronger in men with the serum LDL-cholesterol concentration of 5.0 mmol/l or more than in others. Also, dietary iron intake had a significant association with the disease risk [13]. In eastern Finland, where people have a high fish intake, exceptionally high CHD mortality has been documented. We hypothesized that this paradox could be in part explained by high-mercury content in fish. In the same cohort of men, those who had consumed local non-fatty fish species had elevated hair mercury contents. Controlling for the major CVD risk factors as covariates, dietary intakes of fish, and mercury were associated with significantly increased risk of myocardial infarction and death from CHD [14]. Thus, for the first time, we showed that a high intake of mercury from non-fatty freshwater fish and the consequent accumulation of mercury in the body increase the risk of CHD and mortality.

## Efforts to reduce salt intake

In the 1970s, little was known about risk factors for hypertension besides obesity. Another factor that had been considered decades earlier was high-salt intake, but it was more or less neglected since the unequivocal proof was missing. In addition, data on salt intake at the population level globally was virtually missing since the measurement of habitual salt intake in humans was difficult to obtain through dietary interview. The best method appeared to be the 24-hour urinary sodium excretion, which for obvious reasons was not easy in clinical practice. In the late 1970s, I started to develop increasing interest in salt intake as an important factor for the development of hypertension and its major sequelae stroke. At that time only a few people globally were interested in the health effects of salt, although there were some researchers who considered this important for the development of hypertension. One of them was Prof. Arthur C. Guyton in Jackson, USA, a famous physiologist, whose theories from the 1960s and 1970s related to sodium and blood pressure still hold today [15]. I was lucky to meet him in person in Jackson to discuss these issues.

I was lucky to meet two other young researchers in Finland who were interested in salt intake. Prof. Heikki Karppanen, a pharmacologist had done animal research on the effects of sodium on blood pressure and had an idea to develop a low-sodium salt substitute. In the 1970s he designed two different formulae, both of which are on the market, that reduced sodium intake by ~50% compared with the use of regular NaCl table salt. Prof. Pirjo Pietinen, a nutritionist who had been working in salt assessment studies in the US before returning back to Finland, produced her Ph.D thesis on salt intake, its sources in the Finnish diet, and the assessment of salt intake in dietary studies. I worked with her in this seminal Ph.D dissertation on salt intake in Finland. Both Heikki and Pirjo continued working on issues related to salt throughout their academic careers.

The three of us, supported by some others, started to design more studies around salt intake, and in 1979 we established the North Karelia Salt Project using the same principles as the original North Karelia Project, but now aiming at reducing salt intake in the population [16]. The reasons were obvious: our pilot study showed a very high-salt intake, especially in North Karelian men. Our baseline survey using the 24-hour urinary sodium excretion in a large population survey in 1979 confirmed it; 217 mmol among men and 172 mmol among women [16]. However, we met criticisms from both the medical community and food industry. The food industry in Finland was curious about the salt issue, and gradually the sodium content was reduced in various foods, plus, the inclusion of sodium content was included on the nutrition labelling of foods in Finland. The Ministry of Health and the Finnish Heart Association were also supportive and active in policy development to reduce salt intake in the Finnish diet and established a special rule to label food items “low-salt” in Finland.

The North Karelia Salt Project was also the first, and only, population-based long-term monitoring of salt intake in the world using the gold standard method, the 24-hour urinary sodium excretion measurement, since 1979 [17]. We showed that it is possible to collect 24-hour urine samples in large epidemiological studies successfully, as well as showing that high-salt intake is associated with cardiovascular disease [18, 19] and type 2 diabetes incidence [20]. Our data has shown that a gradual decrease in sodium intake has taken place in Finland, but we are still above the recommended 5 g NaCl/day [17]. Nevertheless, Finland is so far the only country that has such long-term population-based data on salt intake based on proper methodology. World Health Organisation has listed the reduction of salt intake as one of the key elements for the prevention of NCDs by 2025 [21]. It is good to see that the vision I and others had in the 1970s regarding the issue of salt intake reduction and its monitoring was proven right and is now advocated globally.

## Interest in dietary fibre

During the work in North Karelia with a population with a high blood cholesterol level, many approaches were proposed to help people who had this problem. Naturally, the diet was the central issue, as no effective cholesterol-lowering drugs were available at that time. Dr. Denis Burkitt had received honours for his work on virus infection and cancer (Burkitt lymphoma) and was invited to give lectures around the world. In his lecture in Finland he didn’t talk about viruses or cancer but emphasised that dietary fibre intake is highly important for health, which surprised virologists. I developed an interest in the health effects of dietary fibre, especially its potential to lower blood cholesterol. People at the local pharmaceutical company in Finland also became interested in the issue, which resulted in a series of clinical trials and the development of a product based on guar gum, a product that was approved for the management of hypercholesterolaemia, hyperglycaemia and obesity in Finland [22]. An interesting thing happened in the 1980s when I received a request to serve as a witness in the US court in Washington where the government had initiated a court case against a US-based company that produced a guar gum product for weight reduction and used my article as the evidence [23].

## Moving to diabetes and obesity research

In 1980, I took a job in the World Health Organisation in the Western Pacific region as a Medical Officer for Cardiovascular and Metabolic Diseases. The area was huge covering China, all Pacific Island and Australia/New Zealand. Prof. Paul Zimmet had carried out surveys among populations of many Pacific islands and demonstrated the highest prevalence of diabetes at the population level in Nauru, Western Samoa, Fiji, etc. The discussions with Paul led to a long-term collaboration between us that is still ongoing. I started to think that such an epidemic of diabetes may also occur in other areas of the world, which led me to think about how type 2 diabetes (T2D) could be prevented. When I started to speak about the need for the prevention of diabetes [24], I was considered a ridiculous person. Nevertheless, I kept the idea of the primary prevention of T2D in my future research agenda. There were other people getting interested in this issue, which led to a grant application to US National Institutes of Health (NIH) from Sweden and Finland, coordinated by Dr. William Knowler from the famous Pima Indian diabetes research group from Phoenix, USA. Unfortunately, our proposal was not considered for funding by NIH, since we were told that NIH funding for a trial like this may only be possible if it is carried out in the US. Such a trial, Diabetes Prevention Program (DPP) in the US was developed later on [25].

With this background I started to develop a new research protocol for Finland alone. Most of our first grant applications were turned down with a critique such as: “no proper science, just doing what is already known”, “no proper clamp measurements are included to measure insulin resistance”, “physical activity not measured by fitness tests”, etc. A few years later I organized a diabetes prevalence survey as part of a NCD risk factor survey in several areas of Finland in 1992 [26]. From this survey, we identified many people with impaired glucose tolerance (IGT) who were also overweight. They were invited to join the lifestyle intervention trial in order to prevent the progression to T2D. Jointly with Prof. Matti Uusitupa, Professor of Clinical Nutrition from the University of Kuopio (today University of Eastern Finland), Finland I was able to start the Finnish Diabetes Prevention Study (DPS) in 1993 that was gradually expanded to five centres in Finland; Adj. Prof. Jaana Lindström served as the study coordinator. This proof-of-concept randomised controlled trial used a relatively simple lifestyle intervention aiming at improving diet and physical activity. Each clinic had a part-time dietician to advise people allocated to the intensified intervention group [27]. The five main goals of the DPS lifestyle intervention were relatively simple: weight reduction of 5% or more; less than 30% of the energy from fat; less than 10% of the energy from saturated fat; fibre intake of 15 g/1000 kcal (3.6 MJ) or more; and moderate intensity physical activity 30 min/day or more in average.

The DPS was designed to reduce the incidence of T2D by 35% in 5 years. The pre-planned interim analysis of the incidence data was carried out at the point when half of the estimated number of new-onset T2D was reached. We were surprised when the results revealed a 58% risk reduction in the intervention group. Our publication received wide publicity, and it was chosen as the main media topic at the annual American Diabetes Association meeting in 2000, and the article on primary results was published in 2001 [27]. The DPP in the US started to enrol participants to their trial in 1996; the trial design for the lifestyle arm was very similar to the DPS, since we had exchanged information during the development of the protocols. The DPP was planned for six years without any interim analysis on diabetes incidence, but after the DPS publication, the DPP investigators had to analyse their data. The results published in 2002 were striking: 58% risk reduction, exactly the same as in the DPS [25]. The potential for the primary prevention of T2D with a healthy lifestyle was now confirmed in many ethnic groups. We stopped the intervention in the DPS after an average of four years but continued to carry our annual clinical examination of the DPS participants. The long-term post-trial results have demonstrated that the intervention effect sustained and the difference between the original intervention and control groups actually continued to become larger [28]. Thus, the message from the post-trial monitoring is that intervention lasting for a few years may result in long-term, maybe lifelong health benefits. Similar long-term findings have also been published from the Chinese Da Qing IGT lifestyle trial [29]. The interpretation is that lifestyle intervention used in these trials has reduced causative factors of T2D.

The DPS trial has been a rich source for various analyses on health behaviour, modifying effects of diet on T2D risk with diverse genetic backgrounds, studies of biomarkers of diet during the intervention setting, etc. Based on the experiences of the DPS trial, Finland set up the first-ever national T2D prevention programme in the early 2000s [30]. I had the honour to chair the planning of this program. Results from this work have changed the principles related to T2D in primary health care significantly. Early results showed that the one-year intervention reduced the risk of T2D in high-risk individuals; the risk reduction correlated directly with weight reduction [31]. Recently, we have provided long-term follow-up of the high-risk cohort and showed that in individuals who lost weight 2.5% or more during the first year, the 7-year risk reduction in the incidence of drug-treated T2D was 30–40% compared with those with stable weight [32]. It has been very encouraging to see that in many countries around the world T2D prevention has become an important issue. However, in most countries, the actual prevention implementation has been limited, as preventive medicine in general.

Obstructive sleep apnea (OSA) is an increasing public health problem. We carried out a 1-year RCT to determine whether a very low-calorie diet with supervised lifestyle counselling can be an effective treatment for adults with mild OSA [33]. Bodyweight was reduced by 10.7 kg in the intervention group; the apnea–hypopnea index (AHI), the main objectively measured outcome variable, improved by 40% in the intervention group; and the adjusted odds ratio for having mild OSA was lowered by 76%. All common symptoms related to OSA, and features of quality of life improved after the lifestyle intervention. During a 4-year post-intervention follow-up, the intervention group still had 5.5 kg weight reduction and achieved a 61% reduction in the incidence of progression of the OSA compared with the control group [34].

In 1987, with Prof. Zimmet we designed and organised with the Ministry of Health the first NCD risk factor survey in Mauritius that had reported alarmingly high rates of CVD mortality. Mauritius is a multi-ethnic country; we found a high prevalence of T2D plus the mean serum cholesterol was high in all ethnic groups [35], but obesity was not common. When we searched reasons for these uniformly high metabolic disorders, we identified that the most commonly consumed oil was inexpensive “ration oil” where the main component was palm oil. We were planning to carry out trials with diets comprising various oils in order to see whether changes in the type of oil may improve metabolic parameters. However, the government of Mauritius was active and banned palm oil. In 1992, when we carried out the next population survey and the first nutrition survey in the country, we found out that the serum cholesterol level in the Mauritian population had dropped by 0.8 mmol/l [36]. The estimated intake of saturated fatty acids decreased by ~3.5% of energy intake, and the intake of polyunsaturated fatty acids increased by 5.5% of energy intake. This is one of the best examples of the population approach to control metabolic risk factors and prevent chronic diseases by actions done outside the health sector.

I developed more of an interest in obesity research when diabetes became a part of my research portfolio. When we carried out the North Karelia Project, Finnish people, like in most other countries, were not obese. When I was in charge of the WHO MONICA Project data centre in Helsinki in the 1980s, I realised that central adiposity may be important and first introduced it to the Finnish surveys and then proposed that it needs to be included in other MONICA countries, too [37]. This was the first standardised international comparison of abdominal adiposity. Based on our first Finnish results, we surprisingly found out that young middle-aged men who were smokers had a higher waist circumference than non-smokers [38]. This was confirmed with the larger data from the MONICA Project [39] and later on by many other studies. Smokers often have other unhealthy lifestyle habits that can biologically be expressed in various ways.

## Research in children

Finland has the highest incidence of childhood-onset type 1 diabetes (T1D) globally [40]. While the genetic susceptibility that is mainly associated with genes located in the HLA region on chromosome 6, the environmental factors are poorly understood. In the mid-1980s I set up a national “Childhood Diabetes in Finland” study, a comprehensive family study aiming at evaluating effects of genetic and environmental factors on the T1D risk [41]. Research on diet was an important component of the study. A reduced risk conferred by prolonged breastfeeding was observed [42].This was also the first observational study to show that early introduction of dairy products is independently associated with an increased risk of T1D. Dietary nitrite in children with new T1D was higher than in control children, and in mothers of diabetic children dietary nitrite and nitrate intake was higher than in mothers of control children [43]. These findings have been confirmed by some but not all other studies. An interesting study of 25(OH)D concentrations we carried out using serum samples collected during the first trimester of pregnancy from all Finnish women (Finnish Maternity Cohort). No difference was found in serum 25(OH)D concentrations between mothers whose children later developed T1D, and mothers of non-diabetic children of the same age [44]. The current consensus is that vitamin D deficiency may not be causally related to T1D risk. In sum, the role of nutritional factors as well as other environmental factors in the aetiology of T1D unfortunately still remains uncertain.

Prof. David Barker in the UK presented a hypothesis that the risk CVD and T2D in adults are increased in people born small [45]. Several people tried to dispute this, unsuccessfully. In Helsinki Prof. Johan Eriksson working with me found out that at the University Hospital detailed birth records are archived. He designed a prospective study using information coded from these records and linking them to the national registers for hospital discharges, drug cost reimbursement, and death using the national ID number. We worked with Prof. Barker and confirmed his original observations about CVD [46] and T2D [47]. We also had information from child health and school health examinations giving us an opportunity to assess changes in weight during childhood and were able to show the importance of “catch-up growth” (excess weight gain in children born small) during early childhood for these outcomes [48]. Prof Eriksson has continued this line of research with additional data including genetics and with extensive publications on many other outcomes.

## Risk factors for dementia and Alzheimer’s disease recognised

In the late 1990s, my team initiated a follow-up study of people who had been previously participating in our earlier CV D risk factor surveys when they were middle-aged in order to determine the prevalence of cognitive decline—dementia or milder cognitive impairment. To our surprise, we found that all CVD risk factors, behavioural and biological, measured at mid-life, about 22 years earlier, predicted cognitive impairment at old age [49]. Especially unhealthy diet estimated with a simple diet score was highly predictive; people at the lowest tercile of the diet score at mid-life had 90% higher risk of dementia than those at the highest tercile (healthiest) score [50].

Based on these findings we designed the first multimodal lifestyle intervention trial, the “Finnish Geriatric Intervention Study to Prevent Cognitive Impairment and Disability” (FINGER), which is a RCT consisting of nutritional guidance, exercise, cognitive training, and vascular risk factors management demonstrating that a 2-year multidomain lifestyle intervention had beneficial effects on cognition in participants at increased risk of dementia [51]. In our T2D prevention study, the DPS, we showed that a lower intake of total dietary fat and saturated fatty acids, and physical activity were associated with a better cognitive status during a long-term follow-up [52]. Also high BMI and waist were associated with worse cognitive performance. While working in Krems, Austria, I set up a similar multidomain intervention RCT than FINGER to prevent cognitive decline in patients suffering from acute stroke who are at high risk of dementia [53].

In the 1980s Norwegian researchers reported that coffee drinking is associated with elevated serum cholesterol [54]. Since Finland has the highest coffee consumption per capita in the world I and my research team developed an interest in the health effects of coffee. First, we confirmed the original finding of cholesterol raising effect of coffee, but we also found out that it was only related to the “pot boiled” or “Turkish/Greek” type of coffee [55]. We then carried out a controlled trial with various types of coffee brewing and confirmed that only pot boiled coffee increases serum cholesterol [56]. After this, Dutch researchers found two lipid-soluble molecules that they named cafestol and kahweol; these molecules cannot pass filter paper commonly used in the coffee preparation [57]. Since the 1980s, paper-filtered coffee has become the most commonly used type of coffee in Finland, not due to our findings, but because this is an easy way to prepare coffee. Once again, not through medical science but a simple technical idea from Ms Melitta Benz in 1908 has been helping public health!

My research on coffee went on. We showed, against the common belief, that CHD incidence and even total mortality were inversely associated with coffee drinking [58]. Soon after that, we published results on the incidence of T2D: the more coffee the lower the risk [59]. We showed that women who drank at least 10 cups of coffee (~1.3 dl/cup) had a 75% lower risk of T2D than those drinking 0–1 cups/day. With over 25 prospective studies today, we can summarise that every cup of coffee lowers the T2D risk by about 10%. We also showed that 4–5 cups of coffee drinking reduced the risk of dementia by about 50% [60], liver cancer by 70% [61], stroke by 40% [62], and confirmed the previous finding of the preventive effect of coffee on Parkinson’s disease [63]. Recently, we carried out the first metabolomics study in people who participated in a trial drinking different amounts of coffee [64]. The coffee industry is supporting many types of scientific research related to coffee, but unfortunately, their support for health sciences is very small. This is typical for all food industry, since their financial interests are elsewhere, not primarily in health issues.

## Crystal ball

It is needless to say that nutrition and diet play a central role in the development and prevention of chronic diseases. There is a continuous need to carry out epidemiological studies since the exposure (nutritional factors), host (people, their life trajectories), and effect modifiers (social and environmental exposures, food manufacturing) are continuously changing. Also, outcomes are changing continuously as I have experienced during my short research career of a few decades; the major public health issues have changed from CVD to T2D and obesity and emerging impairment of cognition.

There have been increasing debates on health effects of various nutrients, foods, and diets. The only way to provide evidence for suggested effects is to carry out observational and interventional studies. Usually, randomised controlled trials (RCTs) will provide the best evidence regarding various exposures. Some aspects of nutrients, foods, and diets can be tested in RCTs, but many issues cannot. RCTs are usually short-term, therefore, observational studies are important. Luckily, increasing numbers of both RCTs and observational studies on nutrients, foods, and diets have been carried out. A new approach is currently being developed: Precision medicine aims at prevention, diagnosis, and treatment strategies that take individual variability into account [65]. It will incorporate individual variability in genes, environment, and lifestyle with the emphasis in personalised genetic profiling for diagnosis and risk assessment. Nutrigenomics is part of this approach. However, we have to be realistic in this development of precision medicine for public health, although our research results have clearly confirmed that the prevention of non-communicable diseases require interventions targeting multiple risk factors simultaneously.

Another important issue regarding nutrients, foods, and diets is surveillance at the population level and in various age, sex, social, and ethnic groups. This will require considerable resources, whether nutritional surveys and/or biomarker studies, that should be provided by national sources. Unfortunately, many countries have no proper nutritional surveillance, mainly due to the lack of funding. Also, biomarker-based surveillance for instance for salt intake using the 24-hour urinary sodium excretion method has only been carried out in Finland.

My own research and the vast body of science globally have pointed out that diet and other lifestyle issues that influence peoples’ health are often the same for many chronic diseases. This fact has stimulated me to evaluate the effects of lifestyle factors for many health and disease outcomes in observational studies. In addition, I have been lucky to be able to apply this knowledge for health promotion and disease prevention using both the RCT design and population-based interventions.
